# Base Editing in Peanut Using CRISPR/nCas9

**DOI:** 10.3389/fgeed.2022.901444

**Published:** 2022-05-12

**Authors:** Anjanasree K. Neelakandan, Binita Subedi, Sy M. Traore, Papias Binagwa, David A. Wright, Guohao He

**Affiliations:** ^1^ Department of Genetics, Development, and Cell Biology, Iowa State University, Ames, IA, United States; ^2^ Department of Agricultural and Environmental Sciences, Tuskegee University, Tuskegee, AL, United States

**Keywords:** gene-editing, base editing, point mutation, high oleic acid, *FAD2* gene, CRISPR/nCas9, deaminase

## Abstract

Peanut (*Arachis hypogaea* L.), an allotetraploid legume of the Fabaceae family, is able to thrive in tropical and subtropical regions and is considered as a promising oil seed crop worldwide. Increasing the content of oleic acid has become one of the major goals in peanut breeding because of health benefits such as reduced blood cholesterol level, antioxidant properties and industrial benefits such as longer shelf life. Genomic sequencing of peanut has provided evidence of homeologous *AhFAD2A* and *AhFAD2B* genes encoding Fatty Acid Desaturase2 (*FAD2*), which are responsible for catalyzing the conversion of monounsaturated oleic acid into polyunsaturated linoleic acid. Research studies demonstrate that mutations resulting in a frameshift or stop codon in an *FAD2* gene leads to higher oleic acid content in oil. In this study, two expression vectors, pDW3873 and pDW3876, were constructed using Cas9 fused to different deaminases, which were tested as tools to induce point mutations in the promoter and the coding sequences of peanut *AhFAD2* genes. Both constructs harbor the single nuclease null variant, nCas9 D10A, to which the PmCDA1 cytosine deaminase was fused to the C-terminal (pDW3873) while rAPOBEC1 deaminase and an uracil glycosylase inhibitor (UGI) were fused to the N-terminal and the C-terminal respectively (pDW3876). Three gRNAs were cloned independently into both constructs and the functionality and efficiency were tested at three target sites in the *AhFAD2* genes. Both constructs displayed base editing activity in which cytosine was replaced by thymine or other bases in the targeted editing window. pDW3873 showed higher efficiency compared to pDW3876 suggesting that the former is a better base editor in peanut. This is an important step forward considering introgression of existing mutations into elite varieties can take up to 15 years making this tool a benefit for peanut breeders, farmers, industry and ultimately for consumers.

## Introduction

Peanut (*Arachis hypogaea* L*.*) is one of the promising grain legumes under the family Fabaceae as it thrives in many regions of the world and is considered a globally important food/oilseed crop due to its oil content, nutritional value, and many industrial uses (Pandey et al., 2014; Gulluoglu et al., 2016). Conventional breeding has made a great contribution in genetic improvement of agronomic traits and many new peanut varieties have been developed globally, however, breeding programs can be time and resource consuming processes. Alternative strategies to accelerate improvements are needed for global food security, broader uses of peanut products and to meet the demands of climate change. Several research studies have identified genes associated with agronomic traits, such as the well characterized *FAD2* genes, the allergen related and disease resistance genes. For instance, in our previous studies, the genes involved in resistance to early leaf spot disease (Gong et al., 2020) and the mildew locus, which is associated with powdery mildew disease of peanut (Traore et al., 2021) have been identified. Development of advanced genome editing tools for studying the function of these genes would provide a robust research platform leading to the improvement and development of peanut lines with novel agronomic traits associated with disease or abiotic stress resistance, productivity and nutritional value.

The clustered regularly interspaced short palindromic repeats (CRISPR) and CRISPR-associated protein 9 (Cas9) system is globally adopted and proven to be a successful genome editing technology in a wide range of plants and animals. While the CRISPR/Cas9 system is appropriate for insertion/deletion (indel) mutations induced through repairing DNA double-stranded breaks (DSBs) resulting from Cas9 nuclease activity, base editing using a disabled nuclease with a deaminase fusion is an approach for targeted nucleotide conversions with reduced complications caused by DSB repair (Azameti and Dauda, 2021). For this approach, fusions of nickase Cas9 (nCas9) or dead Cas9 (dCas9) to deaminases produces highly specific genome editing tools, which yield targeted changes that mimic natural mutations, the systems are easy to employ, and they maintain high on-target efficiencies while reducing off-target modifications to background levels (Ran et al., 2013).

In the base editor generation 3 (BE3) system, APOBEC1 (Apolipoprotein B mRNA editing enzyme, catalytic polypeptide 1), a cytidine deaminase, is fused to the N-terminal end of nCas9, which features a D10A mutation while an uracil glycosylase inhibitor (UGI) is fused to the C-terminal end. Ideally, nCas9 guides the complex to a target and generates a nick in the protospacer region without generating DSBs while the deaminase effectively mediates C to T conversion and UGI suppress N-glycosylase activity which is responsible for uracil base excision and repair, thus increasing base editing efficiency (Schormann et al., 2014; Komor et al., 2016). In general terms, the conversion of cytosine to thymine may lead to changes in amino acid sequence leading to potential disruption or alteration of enzyme activity of the resulting protein (Cong et al., 2013). Site specific base editing was achieved with the APOBEC1 editing system in monocots to include rice, wheat and maize with no observable indels (Zong et al., 2017; Wang et al., 2020), and in another study, base editing efficiency of 15–75% was achieved in animal and plant cells with very few indel mutations (Rees and Liu, 2018). In another example, the Activation Induced Cytidine Deaminase (AID) enzyme, which normally catalyzes hypermutation of deoxycytidine at the immunoglobulin locus in the vertebrate class switch recombination and repair pathway was fused to nCas9. TARGET-AID is a BE3-like system that constitutes an AID from sea lamprey (PmCDA1) tethered to the C-terminal end of nCas9 (Nishida et al., 2016). TARGET-AID was successfully deployed for efficient base editing in rice and tomato (Shimatani et al., 2017), giving a base editing efficiency ranging from 4–90% in rice (Wu et al., 2019). Examples of base editor uses include loss of function mutations targeting specific motifs or amino acids to inactivate or modify enzyme catalytic domains, elimination of start codons (ATG) to abolish translation, prevention of splicing by disruption of intron/exon junctions and changes in codons such as CGA (Arginine), CAA or CAG (glutamine) and TGG (tryptophan) leading to the introduction of premature stop codons (Rees and Liu, 2018). Overall, base editing expands the scope, ability, and efficiency of Cas9 genome editing by the introduction of targeted point mutations and provides a valuable platform for editing many important traits that are determined by single base changes (Li et al., 2017).

**FIGURE 1:**
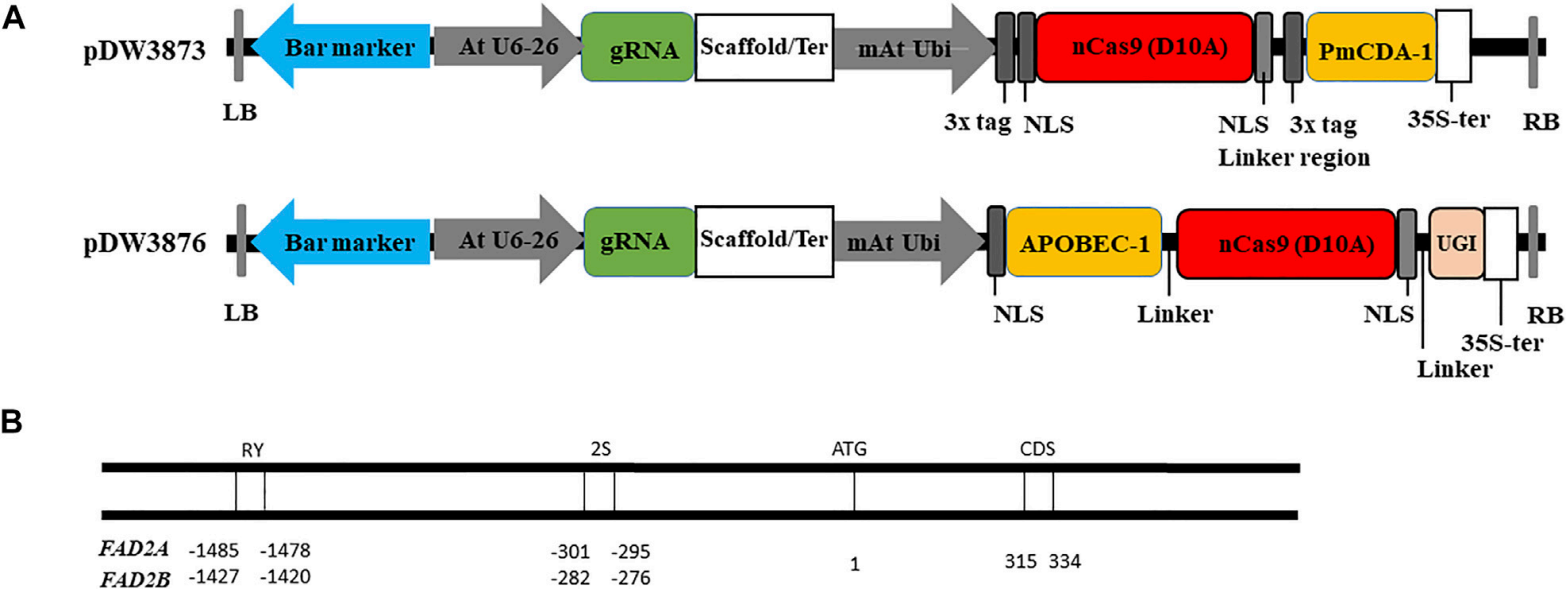
Vectors and targets used for base editing in this study. **(A)**. Construction of vectors with deaminase PmCDA-1 and APOBEC-1. **(B)**. Targets selected in the homeologous gene *FAD2A* and *FAD2B*.

In the present study, two constructs, pDW3873/PmCDA1 and pDW3876/rAPOBEC-1 with UGI were generated (Figure 1) to induce point mutations in the *FAD2* genes as proof of concept in peanut. Their functionality and effectiveness were validated using an *Agrobacterium*-mediated hairy root system and a leaf infiltration method. Successful targeted mutagenesis induced by a CRISPR/Cas9 base editing technology could demonstrate utility for basic and applied research in genes of interest for peanut.

## Materials and Methods

### Plant Material

Peanut genotype GT-C20, a Spanish-type peanut, was used as plant material because there are no known naturally occurring mutations in either *AhFAD2A* or *AhFAD2B* (Yuan et al., 2019). Plants were grown *in-vitro* using well-drained and friable sandy loam soil. The temperature and light conditions used for germination were 15–20°C with a photoperiod of 16 h/day. Seed germinated 7–10 days after sowing.

### Construction of Vectors

For CRISPR/Cas9 base editing constructs, a binary vector was constructed with the following features in the order given: Right border sequence, pVs1 replicon with the *Bsa*I site destroyed, a ColEI replicon, *Npt*I kanamycin resistance gene, and a spectinomycin resistance gene for bacterial selection, left border sequence, a NOS terminator, the bialaphos resistance gene (*Bar*) for *in planta* selection, a 4 × 35 s promoter followed by an *Arabidopsis thaliana* U6 promoter, duel *Bsa*I sites for gRNA oligo insertion, an extended sgRNA scaffold (Dang et al., 2015), U6 terminator, a modified *A. thaliana* Ubiquitin AtUBI promoter, a polylinker for nCas9 fusion cassette insertion, a CaMV 35s terminator then back to the right border sequence.

To produce pDW3873, the binary vector was cut at *Bam*HI and *Spe*I using restriction enzymes following the manufacturer’s instructions then a nCas9 D10A in-frame fusion to the Activation Induced Cytidine Deaminase (AID) enzyme from sea lamprey *(PmCDA1)* cassette was cloned in. The features are a *Bam*HI restriction site, ATG start site, 3x FLAG tag, SV40 NLS, nCas9 D10A, a 104 amino acid linker that includes a second SV40 NLS and a 3xFLAG tag, the PmCDA1 then a stop codon and a *Spe*I restriction site. For construction of pDW3876, the binary vector was cut at *Bam*HI and *Spe*I using restriction enzymes following the manufacturer’s instructions then an APOBEC1 in-frame fusion to nCas9 D10A with an in-frame fusion to UGI was cloned in. The features are a *Bam*HI restriction site, ATG start codon, SV40 NLS, (*Rattus norvegicus*) rat APOBEC1 (Apolipoprotein B mRNA editing enzyme, catalytic polypeptide 1) CDA (rAPOBEC1), 17 amino acid linker, nCas9 D10A, a 16 amino acid linker that includes a 16 amino acid nucleoplasmin NLS, bacteriophage PBS2 from *Bacillus subtilis* uracil glycosylase inhibitor (UGI) then a stop codon and a *Spe*I restriction site. Both vectors were sequence verified prior to use and the deaminase, nCas9 and UGI sequences were codon optimized for dicot expression. Vector designs are illustrated in Figure 1 and full vector sequences are available in the Supplementary File.

Three gRNA target oligos specific to potential *cis*-regulatory elements (RY and 2S seed protein elements) and the coding region (CDS) of both *FAD2* genes were designed using the Cas-Designer tool (Park et al., 2015; CGAT, http://cbc.gdcb.iastate.edu/cgat). Oligonucleotide pairs were synthesized at the Iowa State University DNA facility and were annealed after phosphorylation, to generate sticky ends that correspond to the overhangs generated by *Bsa*I restriction enzyme digestion of expression vectors (pDW3873, pDW3876). The sequences of the guide RNAs are provided in Table 1. The coding region gRNA was inserted into the vectors at the double *Bsa*I cut sites using Gibson Assembly while gRNAs targeting the *cis*-regulatory elements were cloned into vectors using Golden Gate Ligation. Resulting constructs were sequenced to confirm the insertion and correct sequence of the gRNA oligos. Plasmids with a gRNA targeting RY, 2S, and CDS were incorporated into *Agrobacterium rhizogenes* strain K599 for hairy root transformation.

**TABLE 1:** Genomic target site, gRNA and PCR primer information used in hairy root transformation.

Target	Target site	gRNA sequence (5′–3′)	Primer used for PCR (5′–3′)	
			FAD2A	FAD2B
RY	CATGCATG	GATAACATCAACATGCATGCT	F: GTCCTCAAATAGCTCGACTG	F: GAATGAGGATGGGGACCAATATTC
			R: AGGGCCCAGAAGCAATTATGATAC	R: AGGGCCCAGAAGCAATTACTAATG
2S	CAAACAC	GCACCAATTTCCAAACACATG	F: TTGAAGCAAAGGGGTGAGGTTTTC	F: GAAGTAAGGGTTGGTGAAGTTTTC
			R: CAAGTCAATAATCAGTAATCTAATG	R: GCACTACTACAAAGCTAATGGTTC
CDS	CCATGCCTTCAGCAAGTACC	CCATGCCTTCAGCAAGTACC	F: TTACTGATTATTGACTTGCT	F: GAACCATTAGCTTTGTAGTAGTG
			R: CAGAACTTGTTCTTGTACCAAT	R: CAGAACTTGTTCTTGTACCAAT

### Hairy Root Transformation

Sterilized GT-C20 seeds were germinated on ½ MS liquid medium under sterile conditions and grown for approximately 1 week. The embryo roots and lower hypocotyl were cut from seedlings then the remaining upper portion of each seedling was used as explants for hairy root transformation following the modified protocol previously described by Yuan et al. (2019). Briefly, *A. rhizogenes* was streaked on solid LB supplemented with 50 mg/L kanamycin and grown at 28°C overnight. *A. rhizogenes* cells were scraped from the plate and resuspended in 6 ml of ½ MS liquid at the OD of 0.6. Explants were dipped into the *A. rhizogenes* solutions and incubated for 20 min with occasional inverting. After incubation, explants were transferred to ½ MS plates for co-cultivation in the dark at room temperature for 2 days. After co-cultivation, explants were transferred to ½ MS plates supplemented with 300 mg/L timentin and cultured under fluorescent lights at room temperature with a 16-h photoperiod. After 1.5–2 weeks, the induced adventitious hairy roots were harvested from selective media for DNA extraction using the Soltis Lab CTAB DNA Extraction Protocol (Doyle and Doyle, 1987; Cullings, 1992).

### Validation of Mutagenesis

PCR analysis of extracted DNA samples was performed to amplify an approximate 500 bp amplicon bearing the gRNA targeted sites from the *FAD*2*A* and *FAD2B* genes individually. The amplified PCR products were sequenced at the Iowa State University DNA facility and sequencing results were analyzed for mutations using the MEGA7 software package (Kumar et al., 2016).

### Evaluation of the Efficiency of the Edits

Overall editing efficiency was calculated as a percentage of the total number of amplicons with edits from transformed roots screened in hairy root experiments. Editing efficiency of single homeolog events was determined as a percentage of the total number of amplicons with edits in *AhFAD2A* or *AhFAD2B* in the hairy root experiments, while the editing efficiency of dual homeolog events was determined using the number of amplicons in which both *AhFAD2A* and *AhFAD2B* were edited.

## Results

This study was conducted to establish an efficient base editing system in peanut. Three different gRNAs were designed based on the coding region and the promoter of the *AhFAD2* genes (Table 1). The designed gRNAs were cloned separately into the base editing binary vectors pDW3873 and pDW3876 containing a deaminase fused to the C terminal or N terminal of nCas9, respectively. The efficiency of resulting recombinant constructs was tested by a hairy root transformation system using *A. rhizogenes* strain K599. Genomic DNA was isolated from inoculated hairy root samples then PCR amplified using FAD2A or FAD2B target region specific primers (Table 1). The PCR products were sequenced and then analyzed using MEGA7.

### Mutations Detected by Base Editing

Results from base editing experiments showed that the target-activation-induced cytidine deaminase (TARGET-AID) base editor containing PmCDA1 (pDW3873) generated better overall editing efficiency, and specifically higher base editing frequencies around the RY and 2S elements as compared to the rAPOBEC1 BE3 base editing system (pDW3876) (Tables 2). Around the RY element, the overall editing efficiency was 34.6% for pDW3873/RY as compared to 20% for pDW3876/RY and the editing frequency was about two times higher at the *FAD2A* locus as compared to the *FAD2B* (Table 2). Furthermore, the pDW3876/RY base editor produced a higher overall indel frequency (16.0%) as compared to corresponding base editing rate (4.0%) around RY element, whereas pDW3873/RY gave a lower percentage of undesirable indel byproducts (7.7%) and a higher percentage of expected base substitutions (26.9%) at the same site (Table 2). The base editing efficiency was similar (26.9%) around the distal RY and proximal 2S *cis*-elements with the pDW3873/RY and pDW3873/2S constructs, but the indel percentage was higher (15.4%) at 2S. Comparatively, the pDW3876/RY construct resulted in a relatively higher editing frequency around the distal RY element (4.0%) as compared to the pDW3876/2S at the proximal 2S motif, where no edited bases were detected in either *FAD2* genes in the samples analyzed (Table 2).

**TABLE 2:** Evaluation of base editing efficiency using two constructs in hairy root transformation assay.

Construct	No of samples screened	Overall editing efficiency (No of edits with %)	Editing efficiency of both base edits and indels (edited number and percentage)			Editing efficiency of either base edits or indels	
			In FAD2A^a^	In FAD2B^a^	In both FAD2A and FAD2B^b^	Base edits in FAD2A or FAD2B	Indels in FAD2A or FAD2B
pDW3873/RY (nCas9::PmCDA1)	26	9 (34.6%)	7/24 (29.2%)	4/25 (16%)	2 (7.7%)	7 (26.9%)	2 (7.7%)
pDW3876/RY (rAPOBEC1::nCas9::UGI)	25	5 (20%)	3/20 (15%)	2/25 (8%)	0 (0%)	1 (4.0%)	4 (16.0%)
pDW3873/2S (nCas9::PmCDA1)	26	11 (42.3%)	7/18 (38.9%)	8/24 (33.3%)	4 (15.4%)	7 (26.9%)	4 (15.4%)
pDW3876/2S (rAPOBEC1::nCas9::UGI)	28	0 (%)	0 (0%)	0 (0%)	0 (0%)	0 (0%)	0 (0%)
pDW3873/CDS (nCas9::PmCDA1)	11	3 (27%)	3 (27%)	0 (0%)	0 (0%)	2 (18%)	1 (9.1%)

a The percentage of amplicons with edit in total amplicons from FAD2A or FAD2B.

b The percentage of amplicons with both FAD2A and FAD2B edits in total amplicons from FAD2A and FAD2B.

**FIGURE 2:**
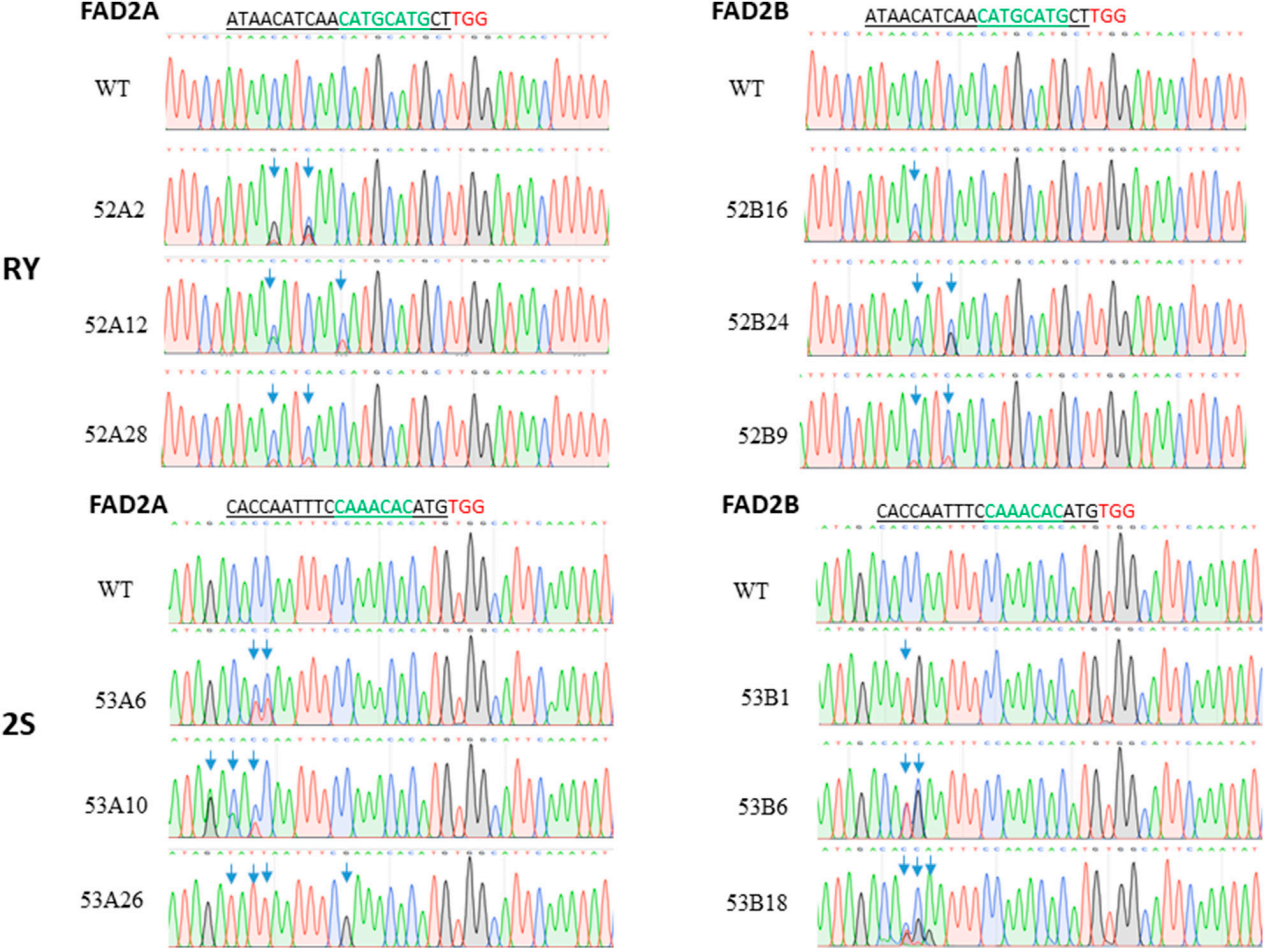
Base editing at the targets RY and 2S. The red letters refer to PAM, the green letters indicated the target, underlined letters are the gRNA sequence and the boxed area represents the observed mutation window.

**FIGURE 3:**
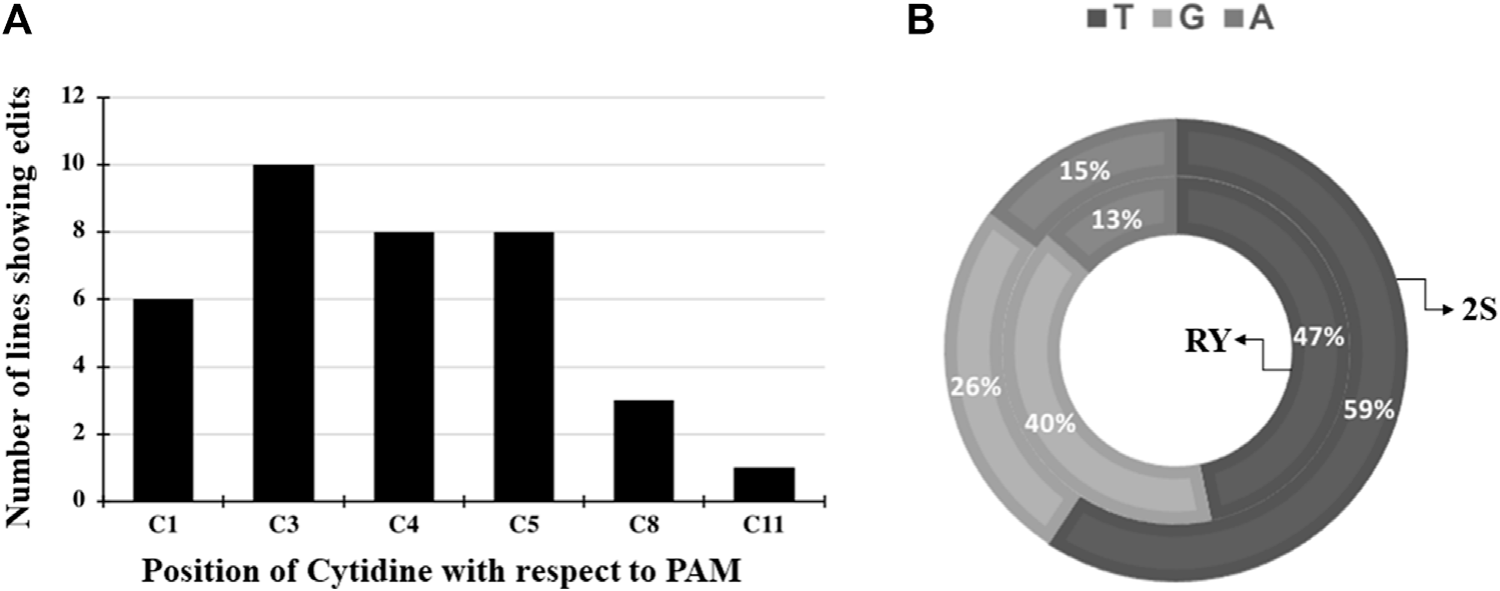
Base editing induced conversion of cytidine to other bases at the RY and 2S targets **(A)** Combined base editing frequencies at different cytidine positions for both RY and 2S targets using pDW3873/PmCDA1; **(B)** Base substitution of C to T, G, A rates at the RY or 2S target by pDW3873/PmCDA1.

Three independent cytidines of the protospacer around the RY motif and four cytidines at the 2S site were found to be modifiable (Figure 2). Rarely, bases flanking the protospacer target window were also substituted as in line 53A10, where a G to A point mutation was observed (Figure 2). Taken together, a higher base editing frequency was observed for cytosine in positions 3 to 5 with respect to the PAM (positions 21–23) which constituted 70% of the total point mutations and the editing window ranged from positions 1 to 11 for PmCDA1 (Figure 3A). Indels and base edits comprised of C to T (47 and 59%), C to G (40 and 26%) and C to A (13 and 15%) were observed around RY and 2S seed protein elements, respectively (Figure 3B).

**FIGURE 4:**
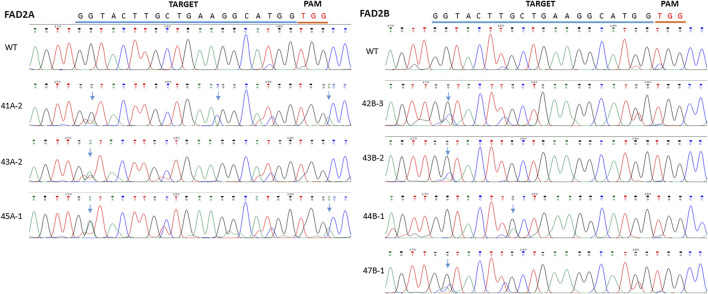
Comparison of mutations between different inoculated samples with vector pDW3873 at targets of *FAD2A* and *FAD2B* using hairy root transformation for the CDS target. The blue line refers to gRNA and the red line indicates PAM. There was one base change (G>A) at the base position 2 within the target, one insertion (C) occurring between the position 13 and 14, and another insertion (A) induced just next to the PAM in *FAD2A*, while two potential base changes (G>C or A) at position 2 and (G>A) at position 8 in the target of *FAD2B*.

To further test the efficiency of base editing in peanut, the coding region of *FAD2* gene was targeted with the vector pDW3873/CDS containing a coding region specific gRNA and efficiency was assayed using hairy root transformation and PCR analysis. Mutations induced by the pDW3873/CDS in *FAD2A* showed a single G to A substitution (18%) at the base position 2 of the protospacer from the PAM. An insertion (A) was detected between G and C right after the PAM and an C was inserted between the position 13 (A) and 14 (G), suggesting indel formation (Figure 4). Although the same position 2 did not convert to A in *FAD2B*, however, multiple peaks (G, A, C) were detected in the sequencing chromatograms. Multiple peaks (G, A) were also detected at the position 8. Besides mutations in the target window, some point mutations were observed in the upstream and downstream sequences flanking the target.

## Discussion

CRISPR/Cas9 mediated gene editing has led to a whole new era for genetic improvement in the field of plant genomics and biotechnology. This system has worked precisely and effectively in many crop species; proving to be a valuable tool for gene functional studies and crop improvement. The popularity of CRISPR/Cas9 technology is increasing as more uses and tools are developed, but to our knowledge, there are no reports of the application of base editing technology in peanut. In this study, we explored the potential application CRISPR/Cas9 mediated based editing technology in peanut, which could lead to improvements in nutritional quality, disease resistance, abiotic stress resistance and improved health-related aspects such as allergenicity and oil quality.

Our results confirm the editing ability of both construct configurations that were tested in peanut, however, pDW3873/PmCDA1/nCas9 had higher editing efficiency than pDW3876/rAPOBEC1/nCas9/UGI. In this study, the pDW3873/PmCDA1 construct base editing efficiency was 26.9% at both the RY and 2S targets (Table 2). In Contrast, pDW3876/rAPOBEC1 editing efficiencies of 4.0 and 0% were observed at the RY and 2S targets, respectively, suggesting that this construct induces relatively low efficiency base editing in peanut (Table 2). It should be noted that rAPOBEC1 mediated base editing is known to be dependent on DNA sequence context and methylation patterns (Wang et al., 2018), which might be responsible for the observed differences in editing efficiency at RY and 2S sites as well as the overall lower editing efficiency compared to the PmCDA1 construct. In contrast, activation induced deaminases like PmCDA1 are reportedly neutral to these factors (Rees and Liu, 2018), which might be responsible for the consistently higher base editing rates at the promoter RY and 2S targets and the coding region for pDW3873/PmCDA1. The rAPOBEC1 base editing system with a low editing efficiency in peanut genome environment suggested that there is a need to test functionality and efficiency of new constructs before general application in a new species (Kim et al., 2017; Wu et al., 2019). Additionally, when comparing the editing efficiency in the promoter and CDS regions of the *FAD2* genes individually, more mutations were induced in *AhFAD2A* than in *AhFAD2B*, 29.2 *vs*. 16% respectively at RY and 38.9 *vs*. 33.3% respectively at 2S and 27% *vs*. 0% respectively in the coding region (Table 2). Furthermore, multiple peaks at cytosine(s) in the sequencing chromatograms were observed in *AhFAD2B*, indicating multiple alleles formed in different tissues during the tests, but multiple peaks were rarely seen in *AhFAD2A* (Figure 4).

The predicted base editing window in this study covered positions 1 to 11 on the protospacer (from 5′-3′) for both base editing constructs and the cytosines at positions 3 to 8 were most likely to be substituted under the test conditions. This result is slightly different from base editor testing in other plant species. For instance, Zong et al. (2017) have shown an editing window spanning position 3 to 9 using rAPOBEC1 fused with nCas9 and UGI, while Wu et al. (2019) reported an editing window spanning positions 1 to 7 using the PmCDA1 fused to UGI in rice. In their construct configurations, indels were observed on the protospacer at relatively high rates. Since the protospacer is nicked by nCas9, DSBs may be created, which may lead to indel formation instead of point mutations. Inclusion of UGI and the bacteriophage Mu GAM gene modules in the vector is reported to help minimize this issue (Komor et al., 2017). However, in our study, rAPOBEC1 paired with nCas9 and UGI did not reduced the rate of indels compared to PmCDA1 paired with nCas9 and without UGI. Another important observation about the constructs in our study was that editing of regions flanking the protospacer was relatively low compared to the distal region. These observed edits could be explained by the length of the linker associating the nCas9 and the cytosine deaminase. For instance, in the pDW3873/PmCDA1 construct, the linker associating nCas9 and PmCDA1 is 104 amino acids. Construction of new vectors with reduced linker length may narrow the base editing window to improve the accuracy and efficiency of future constructs.

Additional Cas9 fusions to deaminases such as human APOBEC3A (Zong et al., 2018) and Adenine Base Editors (ABE; Kang et al., 2018; Li et al., 2018) have been shown to have altered editing windows, sequence preference independence and reduced indel formation rates, while use of other Cas nucleases, which recognize different PAMs, have broadened the number of targets in a genome (Zong et al., 2017; Wu et al., 2019). The above nucleases and deaminases are candidates for future testing in peanut in an effort to identify other base editing systems with higher base editing efficiency, fewer indels, and a narrower target window.

## Conclusion

In this study, we demonstrated base editing activity for two cytidine deaminases that were fused to nCas9 for the induction of point mutations in peanut. The PmCDA1 fusion showed higher editing efficiency compared to the rAPOBEC1 fusion. The effective editing window spanned position 1 to 11 of the protospacer and expected single base substitutions of C to T constituted the predominant type of edits. Base editing has the advantage of minimizing off-target issues, inducing mutations that mimic natural mutations and targeting a specific nucleotide that could lead either to disruption, restoration or amino acid change in a protein or alteration of DNA in a genetic regulatory element. Integrating these approaches into existing tool sets will help to generate new peanut lines with genotypes that may be useful in peanut breeding programs.

## Data Availability

The original contributions presented in the study are included in the article/Supplementary Material, further inquiries can be directed to the corresponding author.
