# Supplementary material for: Base Editing in Peanut Using CRISPR/nCas9
Source: Front Genome Ed. 2022 May 12;4:901444. doi: 10.3389/fgeed.2022.901444 (PMC9133374; doi:10.3389/fgeed.2022.901444)
Supplement: Supplementary file 1 [file DataSheet1.docx]

DNA sequences and maps of two constructs

1. DNA sequences of the construct pDW3873

GAGCTCGATTCActcgagTCGTTGAACAACGGAAACTCGACTTGCCTTCCGCACAATACATCATTTCTTCTTAGCTTTTTTTCTTCTTCTTCGTTCATACAGTTTTTTTTTGTTTATCAGCTTACATTTTCTTGAACCGTAGCTTTCGTTTTCTTCTTTTTAACTTTCCATTCGGAGTTTTTGTATCTTGTTTCATAGTTTGTCCCAGGATTAGAATGATTAGGCATCGAACCTTCAAGAATTTGATTGAATAAAACATCTTCATTCTTAAGATATGAAGATAATCTTCAAAAGGCCCCTGGGAATCTGAAAGAAGAGAAGCAGGCCCATTTATATGGGAAAGAACAATAGTATTTCTTATATAGGCCCATTTAAGTTGAAAACAATCTTCAAAAGTCCCACATCGCTTAGATAAGAAAACGAAGCTGAGTTTATATACAGCTAGAGTCGAAGTAGTGATTGgagaccgttaattaaCCCGGGAGGTCTCGGTTTcAGAGCTAtgctgGAAAcagcaTAGCAAGTTgAAATAAGGCTAGTCCGTTATCAACTTGAAAAAGTGGCACCGAGTCGGTGCTTTTTTTTTTTCTAGACAACAGgaattcAGTCTAGCTCAACAGAGCTTTTAACCCAAATTGGTACAATAGAATACAACTTTAGATCATAATTCTCAAAAGAAAGAGATTCCTTAGCTATTCTATCTGCCACTCCATTTCCTTCTCGGCTTGTATGCACAAGCATAAAATCCTCAAACTTGCTAAGTAGATACTTTATGTCTTGGATAATTGGATTGAGACTTGACAAGCATAACTTTCATGTAACCAAAGACACAAGTTGCTGAGAATCCACCTCAAAAATGATCTTCCTATAATTGAATCGGGATAATGACAGCACAGCCCATCTAAGAGCCTCCACTTCTACTTCCAGCACGCTTCTTACTTTTACCACAGCTCTTGCACCTAACCATAACACCTTCCCTGTATGATCGCGAAGCACCCACCCTAAGCCACATTTTAATCCTTCTGTTGGCCATGCCCCATCAAAGTTGCACTTAACCCAAgATTGTGGTGGAGCTTCCCATGTTTCTCGTCTGTCCCGACGGTGTTGTGGTTGGTGCTTTCCTTACATTCTGAGCCTCTTTCCTTCTAATCCACTCATCTGCATCTTCTTGTGTCCTTACTAATACCTCATTGGTTCCAAATTCCCTCCCTTTAAGCACCAGCTCGTTTCTGTTCTTCCACAGCCTCCCAAGTATCCAAGGGACTAAAGCCTCCACATTCTTCAGATCAGGATATTCTTGTTTAAGATGTTGAACTCTATGGAGGTTTGTATGAACTGATGATCTAGGACCGGATAAGTTCCCTTCTTCATAGCGAACTTATTCAAAGAATGTTTTGTGTATCATTCTTGTTACATTGTTATTAATGAAAAAATATTATTGGTCATTGGACTGAACACGAGTGTTAAATATGGACCAGGCCCCAAATAAGATCCATTGATATATGAATTAAATAACAAGAATAAATCGAGTCACCAAACCACTTGCCTTTTTTAACGAGACTTGTTCACCAACTTGATACAAAAGTCATTATCCTATGCAAATCAATAATCATACAAAAATATCCAATAACACTAAAAAATTAAAAGAAATGGATAATTTCACAATATGTTATACGATAAAGAAGTTACTTTTCCAAGAAATTCACTGATTTTATAAGCCCACTTGCATTAGATAAATGGCAAAAAAAAACAAAAAGGAAAAGAAATAAAGCACGAAGAATcCTAGAAAATACGAAATACGCTTCAATGCAGTGGGACCCACGGTTCAATTATTGCCAATTTTCAGCTCCACCGTATATTTAAAAAATAAAACGATAATGCTAAAAAAATATAAATCGTAACGATCGTTAAATCTCAACGGCTGGATCTTATGACGACCGTTAGAAATTGTGGTTGTCGACGAGTCAGTAATAAACGGCGTCAAAGTGGTTGCAGCCGGCACACACGAGTCGTGTTTATCAACTCAAAGCACAAATActtttCCTCAACCTAAAAATAAGGCAATTAGCCAAAAACAACTTTGCGTGTAAACAACGCTCAATACACGTGTCATTTTATTATTAGCTATTGCTTCACCGCCTTAGCTTTCTCGTGACCTAGTCGTCCTCGTCTTTTCTTCTTCTTCTTCTATAAAACAATACCCAAAGAGCTtTTCTTCTTCACAATTCAGATTTCAATTTCTCAAAATCTTAAAAACTTTCTCTCAATTCTCTCTACCGTGATCAAGGTAAATTTCTGTGTTCCTTATTCTCTCAAAATCTTCGATTTTGTTTTCGTTCGATCCCAATTTCGTATATGTTCTTTGGTTTAGATTCTGTTAATCTTAGATCGAAGACGATTTTCTGGGTTTGATCGTTAGATATCATCTTAATTCTCGATTAGGGTTTCATAGATATCATCCGATTTGTTCAAATAATTTGAGTTTTGTCGAATAATTACTCTTCGATTTGTGATTTCTATCTAaATCTGGTGTTAGTTTCTAGTTTGTGCGATCGAATTTGTCGATTAATCTGAGTTTTTCTGATTAACAGGGATCATCAACAAGTTTGTACAAAAAAGCAGGCTCTTTAAAGTATTTTTACAACAATTACCAACAACAACAAACAACAAACAACATTACAATTACTATTTACAaTTACAAAAAAAggtaccggatccgaaatctgatggactataaggaccacgacggagattacaaggaccatgatattgactacaaggatgacgatgacaaggccgctccaaagaaaaagagaaaggttgggattcacggagttcccgctgccGACAAAAAATACAGCATTGGGTTGGcCATTGGGACAAATAGCGTGGGTTGGGCAGTCATTACAGACGAATACAAGGTTCCTAGCAAGAAGTTCAAGGTGTTGGGCAACACTGACAGGCACTCGATCAAGAAAAATCTCATTGGTGCACTCCTTTTCGATAGTGGGGAGACTGCCGAAGCGACCAGACTGAAAAGGACTGCTAGAAGGCGCTATACCCGGCGTAAGAATCGCATTTGCTACCTCCAGGAGATATTCTCTAACGAAATGGCCAAGGTTGATGACAGCTTCTTTCATaGaTTGGAGGAATCATTCCTCGTCGAGGAAGACAAGAAACACGAGCGGCATCCGATCTTTGGAAACATTGTTGATGAGGTCGCATATCACGAAAAGTACCCCACGATCTACCATCTTCGCAAGAAACTGGTGGATTCAACTGACAAGGCTGATCTGCGGTTGATATACCTCGCACTTGCTCACATGATCAAGTTCCGTGGCCATTTTCTTATTGAGGGtGACCTGAACCCTGATAATTCTGACGTCGATAAGTTGTTCATCCAGCTCGTGCAAACATACAATCAGCTCTTTGAGGAAAACCCTATCAACGCCTCTGGAGTTGATGCCAAAGCGATCCTTTCCGCGAGGCTGTCGAAGAGTAGAAGGCTGGAGAACTTGATCGCCCAACTTCCTGGCGAAAAGAAAAACGGTTTGTTCGGGAATCTCATTGCGCTGAGCTTGGGACTCACGCCAAACTTCAAGTCTAACTTCGACCTCGCAGAGGATGCTAAACTTCAGCTGTCCAAGGATACTTATGATGACGATCTTGACAACCTGTTGGCACAAATCGGTGACCAGTACGCTGATTTGTTCCTCGCTGCCAAGAACCTCAGtGATGCCATACTCCTTTCAGACATCCTCCGCGTGAACACTGAGATTACCAAAGCACCTCTGTCTGCTTCCATGATAAAGCGTTATGATGAGCACCATCAAGACCTCACTCTGTTGAAGGCGTTGGTTAGACAGCAACTCCCAGAGAAGTACAAAGAAATCTTCTTTGACCAATCTAAGAATGGCTACGCCGGATATATTGATGGCGGAGCGTCCCAGGAGGAGTTCTACAAGTTTATCAAACCTATTCTTGAGAAGATGGATGGTACGGAGGAACTCCTTGTTAAACTGAATAGAGAGGATCTCTTGCGCAAGCAACGGACTTTCGACAACGGCAGCATACCGCACCAGATCCATCTCGGAGAGCTTCACGCCATCCTTCGCCGGCAGGAAGATTTCTACCCCTTTCTGAAGGACAACCGTGAGAAGATCGAAAAAATCCTTACTTTCAGAATCCCTTACTATGTCGGTCCACTGGCTAGAGGCAACTCcAGATTTGCTTGGATGACACGCAAGAGTGAGGAAACTATCACCCCTTGGAACTTCGAGGAAGTGGTTGACAAGGGCGCATCGGCTCAAAGTTTCATTGAGAGAATGACTAATTTTGATAAGAACCTTCCGAACGAGAAAGTCCTGCCCAAGCATTCACTCCTTTACGAGTATTTCACAGTGTATAACGAACTTACGAAGGTGAAATACGTTACTGAGGGTATGCGGAAGCCGGCATTCCTGTCGGGGGAACAAAAGAAAGCTATAGTGGATCTGTTGTTCAAAACTAACAGGAAGGTTACCGTCAAGCAGCTTAAGGAGGATTACTTCAAGAAAATAGAATGTTTTGACTCGGTGGAGATCAGTGGCGTTGAAGATAGATTCAACGCTTCACTGGGAACCTACCACGACCTCCTTAAGATCATTAAGGACAAAGATTTCTTGGATAACGAGGAAAATGAGGACATTCTCGAAGATATAGTCCTTACACTGACGTTGTTTGAGGACCGTGAAATGATCGAGGAAAGATTGAAGACCTATGCTCATCTCTTCGACGATAAGGTTATGAAGCAGCTCAAGCGTAGAAGGTACACAGGTTGGGGGCGTCTTTCcAGAAAGCTGATTAATGGCATAAGGGACAAGCAGTCTGGAAAGACGATTCTGGATTTCCTCAAGTCCGACGGCTTCGCCAACCGCAATTTTATGCAGCTTATACACGACGATTCCCTGACTTTCAAAGAGGACATCCAGAAGGCCCAAGTTAGCGGCCAAGGAGATTCACTTCACGAGCATATCGCCAACCTGGCGGGTAGCCCGGCGATCAAGAAAGGGATTCTTCAGACCGTCAAGGTCGTGGATGAGCTGGTGAAAGTTATGGGCCGGCATAAGCCCGAAAACATAGTGATCGAGATGGCCCGTGAAAATCAGACTACCCAAAAAGGACAGAAGAACTCCCGCGAGCGGATGAAAAGGATAGAGGAAGGCATCAAGGAACTTGGAAGCCAGATCCTGAAGGAGCACCCAGTTGAAAACACACAGTTGCAAAATGAGAAGCTCTATCTTTACTATCTCCAAAATGGTAGAGACATGTATGTGGATCAGGAGTTGGACATTAACAGGCTCTCAGATTaCGATGTTGACCATATCGTGCCGCAATCGTTCCTTAAGGACGATAGTATAGATAATAAGGTGCTGACTCGGTcGGATAAAAACCGTGGCAAGTCGGACAATGTTCCCAGTGAGGAAGTTGTGAAGAAGATGAAGAACTaCTGGCGCCAACTGTTGAATGCAAAATTGATCACCCAGAGGAAGTTTGACAACCTCACAAAAGCAGAGCGCGGTGGGCTCAGTGAACTTGATAAAGCTGGATTCATTAAGAGGCAACTTGTTGAGACACGCCAGATTaCGAAGCACGTCGCGCAGATACTGGATTCTAGGATGAACACCAAGTACGACGAGAATGATAAATTGATCAGAGAAGTGAAGGTTATCACATTGAAGTCTAAACTCGTGTCCGATTTTCGCAAAGACTTCCAATTTTATAAGGTTCGGGAGATCAACAATTATCACCATGCACATGATGCTTACCTCAAcGCCGTGGTTGGTACGGCGTTGATTAAGAAATACCCGAAGCTGGAGTCTGAGTTCGTGTACGGGGATTATAAGGTCTACGACGTGAGGAAAATGATCGCTAAGAGTGAGCAGGAAATTGGTaAAgccacCGCGAAGTATTTCTTTTACTCTAACATCATGAACTTCTTCAAGACAGAGATAACGTTGGCAAATGGTGAAATACGTAAGAGGCCTCTCATCGAGACTAACGGTGAAACCGGGGAAATCGTTTGGGATAAAGGGCGGGACTTTGCTACTGTTCGTAAGGTCCTCTCCATGCCGCAAGTGAATATTGTTAAGAAAACAGAGGTCCAGACGGGCGGATTCTCTAAGGAATCCATCCTTCCCAAAAGAAACAGCGACAAGCTGATTGCGAGGAAGAAAGATTGGGACCCTAAGAAATATGGTGGGTTCGATTCTCCAACCGTTGCCTACTCCGTCTTGGTCGTGGCGAAGGTTGAGAAGGGTAAATCTAAGAAACTCAAATCCGTCAAGGAACTCCTTGGGATTACTATCATGGAGAGGTCTTCCTTCGAAAAGAATCCTATCGATTTCCTTGAGGCTAAAGGCTATAAGGAAGTGAAGAAGGATCTCATCATCAAGCTCCCAAAGTACAGCCTGTTTGAGTTGGAAAACGGAAGGAAGCGCATGTTGGCATCAGCTGGAGAACTCCAGAAAGGGAACGAGCTGGCCTTGCCTTCTAAGTATGTGAACTTCCTCTATCTTGCGTCGCACTACGAGAAGCTGAAAGGCAGTCCAGAGGACAACGAACAGAAACAACTTTTTGTTGAGCAACACAAGCATTATCTGGATGAGATTATAGAACAGATCAGCGAGTTCTCAAAAAGAGTCATTCTTGCCGATGCTAACCTCGACAAGGTGCTCTCCGCATACAACAAACACAGAGACAAGCCGATCAGGGAGCAGGCTGAAAATATCATTCATCTCTTCACTCTTACCAACCTGGGTGCCCCCGCGGCATTCAAGTATTTTGATACAACGATTGACCGGAAACGTTACACAAGCACGAAGGAGGTCCTTGATGCGACCCTGATTCATCAAAGCATAACAGGGCTCTATGAGACGAGAATtGATTTGTCACAGCTCGGCGGAGACTCAAGAGCAGACCCTAAgAAaAAGCGAAAAGTGGGaGGTGGCGGTTCTGGAGGtGGaGGtTCCGCCGAATACGTCCGTGCTCTCTTCGACTTCAATGGTAATGACGAGGAAGACCTTCCATTCAAAAAGGGGGACATCTTGCGGATCAGGGATAAGCCCGAGGAACAGTGGTGGAACGCTGAGGACTCTGAAGGTAAGAGAGGCATGATCCCTGTCCCaTACGTCGAGAAATACTCTGGAGATTATAAGGACCACGACGGGGATTATAAAGATCAcGATATtGATTATAAGGAtGACGAtGAcAAGTCCCGAATGACGGACGCCGAGTACGTTAGaATCCACGAGAAACTTGACATCTATACGTTCAAGAAACAATTCTTCAACAATAAAAAGTCAGTGAGCCACCGCTGCTAtGTGTTGTTCGAGTTAAAAAGAAGGGGCGAGCGAAGGGCTTGTTTCTGGGGTTACGCGGTCAACAAGCCACAGTCAGGCACGGAAAGAGGAATACATGCAGAGATTTTCAGcATACGGAAAGTGGAGGAGTACCTGAGAGATAATCCCGGTCAGTTCACTATTAACTGGTAtAGTAGCTGGTCCCCGTGTGCTGATTGtGCAGAAAAGATTCTTGAATGGTATAATCAAGAGCTTCGCGGCAACGGGCATACATTAAAAATCTGGGCTTGTAAaCTTTATTATGAGAAGAACGCTAGGAATCAGATCGGCCTCTGGAACCTCAGAGACAACGGGGTCGGATTGAATGTAATGGTgTCTGAGCACTACCAGTGCTGCCGCAAGATaTTCATCCAGAGCAGCCATAACCAaCTGAATGAAAACAGGTGGTTGGAAAAAACTCTGAAGAGGGCTGAAAAGAGGAGGTCGGAGTTGTCCATaATGATCCAGGTCAAGATACTGCATACGACGAAATCGCCaGCcGTGTCcAGAGGTTCTGGTtaatagactagtGCAAAAATCACCAGTCTCTCTCTACAAATCTATCTCTCTCTATTTTTCTCCAGAATAATGTGTGAGTAGTTCCCAGATAAGGGAATTAGGGTTCTTATAGGGTTTCGCTCATGTGTTGAGCATATAAGAAACCCTTAGTATGTATTTGTATTTGTAAAATACTTCTATCAATAAAATTTCTAATTCCTAAAACCAAAATCCAGTGACCTtgggcccttagtattaagcttagcttgagcttggatcagattgtcgtttcccgccttcagtttaaactatcagtgtttgacaggatatattggcgggtaaacctaagagaaaagagcgtttattagaataatcggatatttaaaagggcgtgaaaaggtttatccgttcgtccatttgtatgtgcatgccaaccacagggttcccctcgggatcaaagtactttaaagtactttaaagtactttaaagtactttgatccaacccctccgctgctatagtgcagtcggcttctgacgttcagtgcagccgtcttctgaaaacgacatgtcgcacaagtcctaagttacgcgacaggctgccgccctgcccttttcctggcgttttcttgtcgcgtgttttagtcgcataaagtagaatacttgcgactagaaccggagacattacgccatgaacaagagcgccgccgctggcctgctgggctatgcccgcgtcagcaccgacgaccaggacttgaccaaccaacgggccgaactgcacgcggccggctgcaccaagctgttttccgagaagatcaccggcaccaggcgcgaccgcccggagctggccaggatgcttgaccacctacgccctggcgacgttgtgacagtgaccaggctagaccgcctggcccgcagcacccgcgacctactggacattgccgagcgcatccaggaggccggcgcgggcctgcgtagcctggcagagccgtgggccgacaccaccacgccggccggccgcatggtgttgaccgtgttcgccggcattgccgagttcgagcgttccctaatcatcgaccgcacccggagcgggcgcgaggccgccaaggcccgaggcgtgaagtttggcccccgccctaccctcaccccggcacagatcgcgcacgcccgcgagctgatcgaccaggaaggccgcaccgtgaaagaggcggctgcactgcttggcgtgcatcgctcgaccctgtaccgcgcacttgagcgcagcgaggaagtgacgcccaccgaggccaggcggcgcggtgccttccgtgaggacgcattgaccgaggccgacgccctggcggccgccgagaatgaacgccaagaggaacaagcatgaaaccgcaccaggacggccaggacgaaccgtttttcattaccgaagagatcgaggcggagatgatcgcggccgggtacgtgttcgagccgcccgcgcacgtctcaaccgtgcggctgcatgaaatcctggccggtttgtctgatgccaagctggcggcctggccggccagcttggccgctgaagaaaccgagcgccgccgtctaaaaaggtgatgtgtatttgagtaaaacagcttgcgtcatgcggtcgctgcgtatatgatgcgatgagtaaataaacaaatacgcaaggggaacgcatgaaggttatcgctgtacttaaccagaaaggcgggtcaggcaagacgaccatcgcaacccatctagcccgcgccctgcaactcgccggggccgatgttctgttagtcgattccgatccccagggcagtgcccgcgattgggcggccgtgcgggaagatcaaccgctaaccgttgtcggcatcgaccgcccgacgattgaccgcgacgtgaaggccatcggccggcgcgacttcgtagtgatcgacggagcgccccaggcggcggacttggctgtgtccgcgatcaaggcagccgacttcgtgctgattccggtgcagccaagcccttacgacatatgggccaccgccgacctggtggagctggttaagcagcgcattgaggtcacggatggaaggctacaagcggcctttgtcgtgtcgcgggcgatcaaaggcacgcgcatcggcggtgaggttgccgaggcgctggccgggtacgagctgcccattcttgagtcccgtatcacgcagcgcgtgagctacccaggcactgccgccgccggcacaaccgttcttgaatcagaacccgagggcgacgctgcccgcgaggtccaggcgctggccgctgaaattaaatcaaaactcatttgagttaatgaggtaaagagaaaatgagcaaaagcacaaacacgctaagtgccggccgtccgagcgcacgcagcagcaaggctgcaacgttggccagcctggcagacacgccagccatgaagcgggtcaactttcagttgccggcggaggatcacaccaagctgaagatgtacgcggtacgccaaggcaagaccattaccgagctgctatctgaatacatcgcgcagctaccagagtaaatgagcaaatgaataaatgagtagatgaattttagcggctaaaggaggcggcatggaaaatcaagaacaaccaggcaccgacgccgtggaatgccccatgtgtggaggaacgggcggttggccaggcgtaagcggctgggttgtctgccggccctgcaatggcactggaacccccaagcccgaggaatcggcgtgagcggtcgcaaaccatccggcccggtacaaatcggcgcggcgctgggtgatgacctggtggagaagttgaaggccgcgcaggccgcccagcggcaacgcatcgaggcagaagcacgccccggtgaatcgtggcaagcggccgctgatcgaatccgcaaagaatcccggcaaccgccggcagccggtgcgccgtcgattaggaagccgcccaagggcgacgagcaaccagattttttcgttccgatgctctatgacgtgggcacccgcgatagtcgcagcatcatggacgtggccgttttccgtctgtcgaagcgtgaccgacgagctggcgaggtgatccgctacgagcttccagacgggcacgtagaggtttccgcagggccggccggcatggccagtgtgtgggattacgacctggtactgatggcggtttcccatctaaccgaatccatgaaccgataccgggaagggaagggagacaagcccggccgcgtgttccgtccacacgttgcggacgtactcaagttctgccggcgagccgatggcggaaagcagaaagacgacctggtagaaacctgcattcggttaaacaccacgcacgttgccatgcagcgtacgaagaaggccaagaacggccgcctggtgacggtatccgagggtgaagccttgattagccgctacaagatcgtaaagagcgaaaccgggcggccggagtacatcgagatcgagctagctgattggatgtaccgcgagatcacagaaggcaagaacccggacgtgctgacggttcaccccgattactttttgatcgatcccggcatcggccgttttctctaccgcctggcacgccgcgccgcaggcaaggcagaagccagatggttgttcaagacgatctacgaacgcagtggcagcgccggagagttcaagaagttctgtttcaccgtgcgcaagctgatcgggtcaaatgacctgccggagtacgatttgaaggaggaggcggggcaggctggcccgatcctagtcatgcgctaccgcaacctgatcgagggcgaagcatccgccggttcctaatgtacggagcagatgctagggcaaattgccctagcaggggaaaaaggtcgaaaacactctttcctgtggatagcacgtacattgggaacccaaagccgtacattgggaaccggaacccgtacattgggaacccaaagccgtacattgggaaccggtcacacatgtaagtgactgatataaaagagaaaaaaggcgatttttccgcctaaaactctttaaaacttattaaaactcttaaaacccgcctggcctgtgcataactgtctggccagcgcacagccgaagagctgcaaaaagcgcctacccttcggtcgctgcgctccctacgccccgccgcttcgcgtcggcctatcgcggccgctggccgctcaaaaatggctggcctacggccaggcaatctaccagggcgcggacaagccgcgccgtcgccactcgaccgccggcgcccacatcaaggcaccctgcctcgcgcgtttcggtgatgacggtgaaaacctctgacacatgcagctcccggagacggtcacagcttgtctgtaagcggatgccgggagcagacaagcccgtcagggcgcgtcagcgggtgttggcgggtgtcggggcgcagccatgacccagtcacgtagcgatagcggagtgtatactggcttaactatgcggcatcagagcagattgtactgagagtgcaccatatgcggtgtgaaataccgcacagatgcgtaaggagaaaataccgcatcaggcgctcttccgcttcctcgctcactgactcgctgcgctcggtcgttcggctgcggcgagcggtatcagctcactcaaaggcggtaatacggttatccacagaatcaggggataacgcaggaaagaacatgtgagcaaaaggccagcaaaaggccaggaaccgtaaaaaggccgcgttgctggcgtttttccataggctccgcccccctgacgagcatcacaaaaatcgacgctcaagtcagaggtggcgaaacccgacaggactataaagataccaggcgtttccccctggaagctccctcgtgcgctctcctgttccgaccctgccgcttaccggatacctgtccgcctttctcccttcgggaagcgtggcgctttctcatagctcacgctgtaggtatctcagttcggtgtaggtcgttcgctccaagctgggctgtgtgcacgaaccccccgttcagcccgaccgctgcgccttatccggtaactatcgtcttgagtccaacccggtaagacacgacttatcgccactggcagcagccactggtaacaggattagcagagcgaggtatgtaggcggtgctacagagttcttgaagtggtggcctaactacggctacactagaaggacagtatttggtatctgcgctctgctgaagccagttaccttcggaaaaagagttggtagctcttgatccggcaaacaaaccaccgctggtagcggtggtttttttgtttgcaagcagcagattacgcgcagaaaaaaaggatctcaagaagatcctttgatcttttctacggggtctgacgctcagtggaacgaaaactcacgttaagggattttggtcatgGtTCTGAGATTATCAAAAAGGATCTTCACCTAGATCCTTTTAAATTAAAAATGAAGTTTTAAATCAATCTAAAGTATATATGTGTAACATTGGTCTAGTGATTAGAAAAACTCATCGAGCATCAAATGAAACTGCAATTTATTCATATCAGGATTATCAATACCATATTTTTGAAAAAGCCGTTTCTGTAATGAAGGAGAAAACTCACCGAGGCAGTTCCATAGGATCGCAAGATCCTGGTATCGGTCTGCGATTCCGACTCGTCCAACATCAATACAACCTATTAATTTCCCCTCGTCAAAAATAAGGTTATCAAGTGAGAAATCACCATGAGTGACGACTGAATCCGGTGAGAATGGCAAAAGTTTATGCATTTCTTTCCAGACTTGTTCAACAGGCCAGCCATTACGCTCGTCATCAAAATCACTCGCATCAACCAAACCGTTATTCATTCGTGATTGCGCCTGAGCGAGACGAAATACGCGGTCGCTGTTAAAAGGACAATTACAAACAGGAATCGAATGCAACCGGCGCAGGAACACTGCTAGCGCATCAACAATATTTTCACCGCTATCCGGATATTCTTCTAATACCTGGAATGCTGTTTTTCCGGGGATCGCAGTGGTGAGTAACCATGCATCATCAGGAGTACGGATAAAATGCTTGATGGTCGGAAGAGGCATAAATTCCGTCAGCCAGTTTAGTCTGACCATCTCATCTGTAACATCATTGGCAACGCTACCTTTGCCATGTTTCAGAAACAACTCTGGCGCATCGGGCTTCCCATACAAGCGATAGATTGTCGCACCTGATTGCCCGACATTATCGCGAGCCCATTTATACCCATATAAATCAGCATCCATGTTGGAATTTAATCGCGGCCTGCTGCAAGACGTTTCCCGTTGAATATGGCTCATAACACCCCTTGTATTACTGTTTATGTAAGCAGACAGTTTTATTGTTCATGATGATATATTTTTATCTTGTGCAATGTAACATCAGAGATTTTGAGACACAACGTGGCTTTCGGAAcatgatatatctcccaatttgtgtagggcttattatgcacgcttaaaaataataaaagcagacttgacctgatagtttggctgtgagcaattatgtgcttagtgcatctaacgcttgagttaagccgcgccgcgaagcggcgtcggcttgaacgaatttctagctagacattatttgccgactaccttggtgatctcgcctttcacgtagtggacaaattcttccaactgatctgcgcgcgaggccaagcgatcttcttcttgtccaagataagcctgtctagcttcaagtatgacgggctgatactgggccggcaggcgctccattgcccagtcggcagcgacatccttcggcgcgattttgccggttactgcgctgtaccaaatgcgggacaacgtaagcactacatttcgctcatcgccagcccagtcgggcggcgagttccatagcgttaaggtttcatttagcgcctcaaatagatcctgttcaggaaccggatcaaagagttcctccgccgctggacctaccaaggcaacgctatgttctcttgcttttgtcagcaagatagccagatcaatgtcgatcgtggctggctcgaagatacctgcaagaatgtcattgcgctgccattctccaaattgcagttcgcgcttagctggataacgccacggaatgatgtcgtcgtgcacaacaatggtgacttctacagcgcggagaatctcgctctctccaggggaagccgaagtttccaaaaggtcgttgatcaaagctcgccgcgttgtttcatcaagccttacggtcaccgtaaccagcaaatcaatatcactgtgtggcttcaggccgccatccactgcggagccgtacaaatgtacggccagcaacgtcggttcgagatggcgctcgatgacgccaactacctctgatagttgagtcgatacttcggcgatcaccgcttcccccatgatgtttaactttgttttagggcgactgccctgctgcgtaacatcgttgctgctccataacatcaaacatcgacccacggcgtaacgcgcttgctgcttggatgcccgaggcatagactgtaccccaaaaaaacagtcataacaagccatgaaaaccgccactgcgccgttaccaccgctgcgttcggtcaaggttctggaccagttgcgtgagcgcatacgctacttgcattacagcttacgaaccgaacaggcttatgtccactgggttcgtgcccgaattgatcacaggcagcaacgctctgtcatcgttacaatcaacatgctaccctccgcgagatcatccgtgtttcaaacccggcagcttagttgccgttcttccgaatagcatcggtaacatgagcaaagtctgccgccttacaacggctctcccgctgacgccgtcccggactgatgggctgcctgtatcgagtggtgattttgtgccgagctgccggtcggggagctgttggctggctggtggcaggatatattgtggtgtaaacaaattgacgcttagacaacttaataacacattgcggacgtttttaatgtactgaattaacgccgaattgaattatcagcttgcatgccggtcgatctagtaacatagatgacaccgcgcgcgataatttatcctagtttgcgcgctatattttgttttctatcgcgtattaaatgtataattgcgggactctaatcataaaaacccatctcataaataacgtcatgcattacatgttaattattacatgcttaacgtaattcaacagaaattatatgataatcatcgcaagaccggcaacaggattcaatCTTAAGAAACTTTATTGCCAAATGTTGAACGATGGATCAAATCTCGGTGACTGGCAGGACAGGACGTGGCGGCACCGGCAGGCTGAAGTCCAGCTGCCAGAAACCCACGTCATGCCAGTTCCCGTGCTTGAAGCCGGCCGCCCGCAGCATGCCGCGGGGGGCATATCCGAGCGCCTCGTGCATGCGCACGCTCGGGTCGTTGGGCAGCCCGATGACAGCGACCACGCTCTTGAAGCCCTGTGCCTCCAGGGACTTCAGCAGGTGGGTGTAGAGCGTGGAGCCCAGTCCCGTCCGCTGGTGGCGGGGGGAGACGTACACGGTCGACTCGGCCGTCCAGTCGTAGGCGTTGCGTGCCTTCCAGGGGCCGGCGTAGGCGATGCCGGCGACCTCGCCGTCCACCTCGGCGACGAGCCAGGGATAGCGCTCCCGCAGACGGACGAGGTCGTCCGTCCACTCCTGCGGTTCCTGCGGCTCGGTACGGAAGTTGACCGTGCTTGTCTCGATGTAGTGGTTGACGATGGTGCAGACCGCCGGCATGTCCGCCTCGGTGGCACGGCGGATGTCGGCCGGGCGTCGTTCTGGGCTCATGGTTACTTCCTAATCTATGGTTCCGCGATAATTGTAAATGTAATTGTAATGTTGTTTGTTGTTTGTTGTTGTTGGTAATTGTTGTAAAAATGCTTATACTCCAGCGTGTCCTCTCCAAATGAAATGAACTTCCTTATATAGAGGAAGGGTCTTGCGAAGGATAGTGGGATTGTGCGTCATCCCTTACGTCAGTGGAGATGTCACATCAATCCACTTGCTTTGAAGACGTGGTTGGAACGTCTTCTTTTTCCACGATGCTCCTCGTGGGTGGGGGTCCATCTTTGGGACCACTGTCGGCAGAGAGATCCTGCGAAGGATAGTGGGATTGTGCGTCATCCCTTACGTCAGTGGAGATGTCACATCAATCCACTTGCTTTGAAGACGTGGTTGGAACGTCTTCTTTTTCCACGATGCTCCTCGTGGGTGGGGGTCCATCTTTGGGACCACTGTCGGCAGAGAGATCCTGCGAAGGATAGTGGGATTGTGCGTCATCCCTTACGTCAGTGGAGATGTCACATCAATCCACTTGCTTTGAAGACGTGGTTGGAACGTCTTCTTTTTCCACGATGCTCCTCGTGGGTGGGGGTCCATCTTTGGGACCACTGTCGGCAGAGAGATCCTGCGAAGATAGTGGGATTGTGCGTCATCCCTTACGTCAGTGGAGATGTCACATCAATCCACTTGCTTTGAAGACGTGGTTGGAACGTCTTCTTTTTCCACGATGCTCCTCGTGGGTGGGGGTCCATCTTTGGGACCACTGTCGGCA

1. Map of the pDW3873 construct (dual *Bsa*I cut sites for insertion of gRNA are highlighted in blue)

1. DNA sequences of the construct pDW3876

GAGCTCGATTCActcgagTCGTTGAACAACGGAAACTCGACTTGCCTTCCGCACAATACATCATTTCTTCTTAGCTTTTTTTCTTCTTCTTCGTTCATACAGTTTTTTTTTGTTTATCAGCTTACATTTTCTTGAACCGTAGCTTTCGTTTTCTTCTTTTTAACTTTCCATTCGGAGTTTTTGTATCTTGTTTCATAGTTTGTCCCAGGATTAGAATGATTAGGCATCGAACCTTCAAGAATTTGATTGAATAAAACATCTTCATTCTTAAGATATGAAGATAATCTTCAAAAGGCCCCTGGGAATCTGAAAGAAGAGAAGCAGGCCCATTTATATGGGAAAGAACAATAGTATTTCTTATATAGGCCCATTTAAGTTGAAAACAATCTTCAAAAGTCCCACATCGCTTAGATAAGAAAACGAAGCTGAGTTTATATACAGCTAGAGTCGAAGTAGTGATTGgagaccgttaattaaCCCGGGAGGTCTCGGTTTcAGAGCTAtgctgGAAAcagcaTAGCAAGTTgAAATAAGGCTAGTCCGTTATCAACTTGAAAAAGTGGCACCGAGTCGGTGCTTTTTTTTTTTCTAGACAACAGgaattcAGTCTAGCTCAACAGAGCTTTTAACCCAAATTGGTACAATAGAATACAACTTTAGATCATAATTCTCAAAAGAAAGAGATTCCTTAGCTATTCTATCTGCCACTCCATTTCCTTCTCGGCTTGTATGCACAAGCATAAAATCCTCAAACTTGCTAAGTAGATACTTTATGTCTTGGATAATTGGATTGAGACTTGACAAGCATAACTTTCATGTAACCAAAGACACAAGTTGCTGAGAATCCACCTCAAAAATGATCTTCCTATAATTGAATCGGGATAATGACAGCACAGCCCATCTAAGAGCCTCCACTTCTACTTCCAGCACGCTTCTTACTTTTACCACAGCTCTTGCACCTAACCATAACACCTTCCCTGTATGATCGCGAAGCACCCACCCTAAGCCACATTTTAATCCTTCTGTTGGCCATGCCCCATCAAAGTTGCACTTAACCCAAgATTGTGGTGGAGCTTCCCATGTTTCTCGTCTGTCCCGACGGTGTTGTGGTTGGTGCTTTCCTTACATTCTGAGCCTCTTTCCTTCTAATCCACTCATCTGCATCTTCTTGTGTCCTTACTAATACCTCATTGGTTCCAAATTCCCTCCCTTTAAGCACCAGCTCGTTTCTGTTCTTCCACAGCCTCCCAAGTATCCAAGGGACTAAAGCCTCCACATTCTTCAGATCAGGATATTCTTGTTTAAGATGTTGAACTCTATGGAGGTTTGTATGAACTGATGATCTAGGACCGGATAAGTTCCCTTCTTCATAGCGAACTTATTCAAAGAATGTTTTGTGTATCATTCTTGTTACATTGTTATTAATGAAAAAATATTATTGGTCATTGGACTGAACACGAGTGTTAAATATGGACCAGGCCCCAAATAAGATCCATTGATATATGAATTAAATAACAAGAATAAATCGAGTCACCAAACCACTTGCCTTTTTTAACGAGACTTGTTCACCAACTTGATACAAAAGTCATTATCCTATGCAAATCAATAATCATACAAAAATATCCAATAACACTAAAAAATTAAAAGAAATGGATAATTTCACAATATGTTATACGATAAAGAAGTTACTTTTCCAAGAAATTCACTGATTTTATAAGCCCACTTGCATTAGATAAATGGCAAAAAAAAACAAAAAGGAAAAGAAATAAAGCACGAAGAATcCTAGAAAATACGAAATACGCTTCAATGCAGTGGGACCCACGGTTCAATTATTGCCAATTTTCAGCTCCACCGTATATTTAAAAAATAAAACGATAATGCTAAAAAAATATAAATCGTAACGATCGTTAAATCTCAACGGCTGGATCTTATGACGACCGTTAGAAATTGTGGTTGTCGACGAGTCAGTAATAAACGGCGTCAAAGTGGTTGCAGCCGGCACACACGAGTCGTGTTTATCAACTCAAAGCACAAATActtttCCTCAACCTAAAAATAAGGCAATTAGCCAAAAACAACTTTGCGTGTAAACAACGCTCAATACACGTGTCATTTTATTATTAGCTATTGCTTCACCGCCTTAGCTTTCTCGTGACCTAGTCGTCCTCGTCTTTTCTTCTTCTTCTTCTATAAAACAATACCCAAAGAGCTtTTCTTCTTCACAATTCAGATTTCAATTTCTCAAAATCTTAAAAACTTTCTCTCAATTCTCTCTACCGTGATCAAGGTAAATTTCTGTGTTCCTTATTCTCTCAAAATCTTCGATTTTGTTTTCGTTCGATCCCAATTTCGTATATGTTCTTTGGTTTAGATTCTGTTAATCTTAGATCGAAGACGATTTTCTGGGTTTGATCGTTAGATATCATCTTAATTCTCGATTAGGGTTTCATAGATATCATCCGATTTGTTCAAATAATTTGAGTTTTGTCGAATAATTACTCTTCGATTTGTGATTTCTATCTAaATCTGGTGTTAGTTTCTAGTTTGTGCGATCGAATTTGTCGATTAATCTGAGTTTTTCTGATTAACAGGGATCATCAACAAGTTTGTACAAAAAAGCAGGCTCTTTAAAGTATTTTTACAACAATTACCAACAACAACAAACAACAAACAACATTACAATTACTATTTACAaTTACAAAAAAAggtaccggatccgaaatctgATGcCTAAGAAGAAACGGAAGGTgTCTTCCGAGACGGGtCCtGTGGCTGTGGAcCCAACTCTCAGGAGGAGAATCGAGCCCCACGAATTTGAGGTGTTTTTCGACCCCCGTGAACTGCGtAAGGAGACTTGCTTGTTATATGAGATTAACTGGGGGGGAAGGCATAGCATcTGGAGACATACGTCACAGAATACTAACAAGCATGTGGAGGTgAACTTTATCGAGAAATTCACAACTGAGCGCTATTTCTGCCCTAACACGCGCTGTTCCATTACTTGGTTTCTTAGCTGGAGtCCTTGTGGCGAATGTTCAAGAGCCATCACTGAgTTCCTATCCCGCTATCCTCACGTGACTCTATTTATCTACATCGCTCGTCTGTATCACCACGCTGATCCTCGGAATAGACAAGGGCTTCGGGACCTTATTTCCTCAGGTGTCACTATACAGATAATGACGGAGCAGGAATCTGGATATTGCTGGCGCAATTTCGTGAATTATAGCCCTTCAAATGAGGCACATTGGCCCAGATACCCCCACCTTTGGGTGCGCTTGTACGTTCTTGAACTTTATTGCATCATTTTGGGCCTTCCTCCTTGTCTAAACATTCTCAGGAGAAAGCAGCCTCAgTTGACATTCTTCACAATAGCCCTGCAATCGTGTCATTATCAACGCCTTCCCCCACACATCCTGTGGGCTACTGGGTTAAAGTCGGGATCTGAGACACCAGGAACATCCGAGTCTGCCACACCAGAGCTGAAGGACAAAAAATACAGCATTGGGTTGGcCATTGGGACAAATAGCGTGGGTTGGGCAGTCATTACAGACGAATACAAGGTTCCTAGCAAGAAGTTCAAGGTGTTGGGCAACACTGACAGGCACTCGATCAAGAAAAATCTCATTGGTGCACTCCTTTTCGATAGTGGGGAGACTGCCGAAGCGACCAGACTGAAAAGGACTGCTAGAAGGCGCTATACCCGGCGTAAGAATCGCATTTGCTACCTCCAGGAGATATTCTCTAACGAAATGGCCAAGGTTGATGACAGCTTCTTTCATaGaTTGGAGGAATCATTCCTCGTCGAGGAAGACAAGAAACACGAGCGGCATCCGATCTTTGGAAACATTGTTGATGAGGTCGCATATCACGAAAAGTACCCCACGATCTACCATCTTCGCAAGAAACTGGTGGATTCAACTGACAAGGCTGATCTGCGGTTGATATACCTCGCACTTGCTCACATGATCAAGTTCCGTGGCCATTTTCTTATTGAGGGtGACCTGAACCCTGATAATTCTGACGTCGATAAGTTGTTCATCCAGCTCGTGCAAACATACAATCAGCTCTTTGAGGAAAACCCTATCAACGCCTCTGGAGTTGATGCCAAAGCGATCCTTTCCGCGAGGCTGTCGAAGAGTAGAAGGCTGGAGAACTTGATCGCCCAACTTCCTGGCGAAAAGAAAAACGGTTTGTTCGGGAATCTCATTGCGCTGAGCTTGGGACTCACGCCAAACTTCAAGTCTAACTTCGACCTCGCAGAGGATGCTAAACTTCAGCTGTCCAAGGATACTTATGATGACGATCTTGACAACCTGTTGGCACAAATCGGTGACCAGTACGCTGATTTGTTCCTCGCTGCCAAGAACCTCAGtGATGCCATACTCCTTTCAGACATCCTCCGCGTGAACACTGAGATTACCAAAGCACCTCTGTCTGCTTCCATGATAAAGCGTTATGATGAGCACCATCAAGACCTCACTCTGTTGAAGGCGTTGGTTAGACAGCAACTCCCAGAGAAGTACAAAGAAATCTTCTTTGACCAATCTAAGAATGGCTACGCCGGATATATTGATGGCGGAGCGTCCCAGGAGGAGTTCTACAAGTTTATCAAACCTATTCTTGAGAAGATGGATGGTACGGAGGAACTCCTTGTTAAACTGAATAGAGAGGATCTCTTGCGCAAGCAACGGACTTTCGACAACGGCAGCATACCGCACCAGATCCATCTCGGAGAGCTTCACGCCATCCTTCGCCGGCAGGAAGATTTCTACCCCTTTCTGAAGGACAACCGTGAGAAGATCGAAAAAATCCTTACTTTCAGAATCCCTTACTATGTCGGTCCACTGGCTAGAGGCAACTCcAGATTTGCTTGGATGACACGCAAGAGTGAGGAAACTATCACCCCTTGGAACTTCGAGGAAGTGGTTGACAAGGGCGCATCGGCTCAAAGTTTCATTGAGAGAATGACTAATTTTGATAAGAACCTTCCGAACGAGAAAGTCCTGCCCAAGCATTCACTCCTTTACGAGTATTTCACAGTGTATAACGAACTTACGAAGGTGAAATACGTTACTGAGGGTATGCGGAAGCCGGCATTCCTGTCGGGGGAACAAAAGAAAGCTATAGTGGATCTGTTGTTCAAAACTAACAGGAAGGTTACCGTCAAGCAGCTTAAGGAGGATTACTTCAAGAAAATAGAATGTTTTGACTCGGTGGAGATCAGTGGCGTTGAAGATAGATTCAACGCTTCACTGGGAACCTACCACGACCTCCTTAAGATCATTAAGGACAAAGATTTCTTGGATAACGAGGAAAATGAGGACATTCTCGAAGATATAGTCCTTACACTGACGTTGTTTGAGGACCGTGAAATGATCGAGGAAAGATTGAAGACCTATGCTCATCTCTTCGACGATAAGGTTATGAAGCAGCTCAAGCGTAGAAGGTACACAGGTTGGGGGCGTCTTTCcAGAAAGCTGATTAATGGCATAAGGGACAAGCAGTCTGGAAAGACGATTCTGGATTTCCTCAAGTCCGACGGCTTCGCCAACCGCAATTTTATGCAGCTTATACACGACGATTCCCTGACTTTCAAAGAGGACATCCAGAAGGCCCAAGTTAGCGGCCAAGGAGATTCACTTCACGAGCATATCGCCAACCTGGCGGGTAGCCCGGCGATCAAGAAAGGGATTCTTCAGACCGTCAAGGTCGTGGATGAGCTGGTGAAAGTTATGGGCCGGCATAAGCCCGAAAACATAGTGATCGAGATGGCCCGTGAAAATCAGACTACCCAAAAAGGACAGAAGAACTCCCGCGAGCGGATGAAAAGGATAGAGGAAGGCATCAAGGAACTTGGAAGCCAGATCCTGAAGGAGCACCCAGTTGAAAACACACAGTTGCAAAATGAGAAGCTCTATCTTTACTATCTCCAAAATGGTAGAGACATGTATGTGGATCAGGAGTTGGACATTAACAGGCTCTCAGATTaCGATGTTGACCATATCGTGCCGCAATCGTTCCTTAAGGACGATAGTATAGATAATAAGGTGCTGACTCGGTcGGATAAAAACCGTGGCAAGTCGGACAATGTTCCCAGTGAGGAAGTTGTGAAGAAGATGAAGAACTaCTGGCGCCAACTGTTGAATGCAAAATTGATCACCCAGAGGAAGTTTGACAACCTCACAAAAGCAGAGCGCGGTGGGCTCAGTGAACTTGATAAAGCTGGATTCATTAAGAGGCAACTTGTTGAGACACGCCAGATTaCGAAGCACGTCGCGCAGATACTGGATTCTAGGATGAACACCAAGTACGACGAGAATGATAAATTGATCAGAGAAGTGAAGGTTATCACATTGAAGTCTAAACTCGTGTCCGATTTTCGCAAAGACTTCCAATTTTATAAGGTTCGGGAGATCAACAATTATCACCATGCACATGATGCTTACCTCAAcGCCGTGGTTGGTACGGCGTTGATTAAGAAATACCCGAAGCTGGAGTCTGAGTTCGTGTACGGGGATTATAAGGTCTACGACGTGAGGAAAATGATCGCTAAGAGTGAGCAGGAAATTGGTaAAgccacCGCGAAGTATTTCTTTTACTCTAACATCATGAACTTCTTCAAGACAGAGATAACGTTGGCAAATGGTGAAATACGTAAGAGGCCTCTCATCGAGACTAACGGTGAAACCGGGGAAATCGTTTGGGATAAAGGGCGGGACTTTGCTACTGTTCGTAAGGTCCTCTCCATGCCGCAAGTGAATATTGTTAAGAAAACAGAGGTCCAGACGGGCGGATTCTCTAAGGAATCCATCCTTCCCAAAAGAAACAGCGACAAGCTGATTGCGAGGAAGAAAGATTGGGACCCTAAGAAATATGGTGGGTTCGATTCTCCAACCGTTGCCTACTCCGTCTTGGTCGTGGCGAAGGTTGAGAAGGGTAAATCTAAGAAACTCAAATCCGTCAAGGAACTCCTTGGGATTACTATCATGGAGAGGTCTTCCTTCGAAAAGAATCCTATCGATTTCCTTGAGGCTAAAGGCTATAAGGAAGTGAAGAAGGATCTCATCATCAAGCTCCCAAAGTACAGCCTGTTTGAGTTGGAAAACGGAAGGAAGCGCATGTTGGCATCAGCTGGAGAACTCCAGAAAGGGAACGAGCTGGCCTTGCCTTCTAAGTATGTGAACTTCCTCTATCTTGCGTCGCACTACGAGAAGCTGAAAGGCAGTCCAGAGGACAACGAACAGAAACAACTTTTTGTTGAGCAACACAAGCATTATCTGGATGAGATTATAGAACAGATCAGCGAGTTCTCAAAAAGAGTCATTCTTGCCGATGCTAACCTCGACAAGGTGCTCTCCGCATACAACAAACACAGAGACAAGCCGATCAGGGAGCAGGCTGAAAATATCATTCATCTCTTCACTCTTACCAACCTGGGTGCCCCcGCgGCCTTCAAGTATTTCGACACCACCATAGATAGGAAACGCTACACTTCAACTAAGGAGGTCCTTGATGCGACGCTCATCCATCAGAGCATCACGGGCCTTTATGAAACAAGGATtGATCTGAGCCAaCTGGGTGGAGATAAGAGgCCtGCTGCTACTAAGAAAGCGGGTCAAGCGAAGAAGAAGAAGACAAGGGACAGCGGAGGGTCcACAAACCTCTCGGACATCATTGAGAAAGAaACCGGCAAACAGCTAGTTATTCAGGAGTCTATCCTGATGCTGCCAGAGGAGGTCGAAGAGGTTATAGGCAACAAGCCGGAAAGTGACATTCTGGTTCACACGGCATAcGATGAGTCTACAGATGAGAACGTGATGTTGCTTACCTCGGACGCTCCAGAGTATAAACCTTGGGCtCTGGTGATCCAGGATTCAAACGGAGAGAACAAGATAAAGATGCTCTCTGGAGGTTCCCCTAAGAAAAAGAGGAAGGTTtaatagactagtGCAAAAATCACCAGTCTCTCTCTACAAATCTATCTCTCTCTATTTTTCTCCAGAATAATGTGTGAGTAGTTCCCAGATAAGGGAATTAGGGTTCTTATAGGGTTTCGCTCATGTGTTGAGCATATAAGAAACCCTTAGTATGTATTTGTATTTGTAAAATACTTCTATCAATAAAATTTCTAATTCCTAAAACCAAAATCCAGTGACCTtgggcccttagtattaagcttagcttgagcttggatcagattgtcgtttcccgccttcagtttaaactatcagtgtttgacaggatatattggcgggtaaacctaagagaaaagagcgtttattagaataatcggatatttaaaagggcgtgaaaaggtttatccgttcgtccatttgtatgtgcatgccaaccacagggttcccctcgggatcaaagtactttaaagtactttaaagtactttaaagtactttgatccaacccctccgctgctatagtgcagtcggcttctgacgttcagtgcagccgtcttctgaaaacgacatgtcgcacaagtcctaagttacgcgacaggctgccgccctgcccttttcctggcgttttcttgtcgcgtgttttagtcgcataaagtagaatacttgcgactagaaccggagacattacgccatgaacaagagcgccgccgctggcctgctgggctatgcccgcgtcagcaccgacgaccaggacttgaccaaccaacgggccgaactgcacgcggccggctgcaccaagctgttttccgagaagatcaccggcaccaggcgcgaccgcccggagctggccaggatgcttgaccacctacgccctggcgacgttgtgacagtgaccaggctagaccgcctggcccgcagcacccgcgacctactggacattgccgagcgcatccaggaggccggcgcgggcctgcgtagcctggcagagccgtgggccgacaccaccacgccggccggccgcatggtgttgaccgtgttcgccggcattgccgagttcgagcgttccctaatcatcgaccgcacccggagcgggcgcgaggccgccaaggcccgaggcgtgaagtttggcccccgccctaccctcaccccggcacagatcgcgcacgcccgcgagctgatcgaccaggaaggccgcaccgtgaaagaggcggctgcactgcttggcgtgcatcgctcgaccctgtaccgcgcacttgagcgcagcgaggaagtgacgcccaccgaggccaggcggcgcggtgccttccgtgaggacgcattgaccgaggccgacgccctggcggccgccgagaatgaacgccaagaggaacaagcatgaaaccgcaccaggacggccaggacgaaccgtttttcattaccgaagagatcgaggcggagatgatcgcggccgggtacgtgttcgagccgcccgcgcacgtctcaaccgtgcggctgcatgaaatcctggccggtttgtctgatgccaagctggcggcctggccggccagcttggccgctgaagaaaccgagcgccgccgtctaaaaaggtgatgtgtatttgagtaaaacagcttgcgtcatgcggtcgctgcgtatatgatgcgatgagtaaataaacaaatacgcaaggggaacgcatgaaggttatcgctgtacttaaccagaaaggcgggtcaggcaagacgaccatcgcaacccatctagcccgcgccctgcaactcgccggggccgatgttctgttagtcgattccgatccccagggcagtgcccgcgattgggcggccgtgcgggaagatcaaccgctaaccgttgtcggcatcgaccgcccgacgattgaccgcgacgtgaaggccatcggccggcgcgacttcgtagtgatcgacggagcgccccaggcggcggacttggctgtgtccgcgatcaaggcagccgacttcgtgctgattccggtgcagccaagcccttacgacatatgggccaccgccgacctggtggagctggttaagcagcgcattgaggtcacggatggaaggctacaagcggcctttgtcgtgtcgcgggcgatcaaaggcacgcgcatcggcggtgaggttgccgaggcgctggccgggtacgagctgcccattcttgagtcccgtatcacgcagcgcgtgagctacccaggcactgccgccgccggcacaaccgttcttgaatcagaacccgagggcgacgctgcccgcgaggtccaggcgctggccgctgaaattaaatcaaaactcatttgagttaatgaggtaaagagaaaatgagcaaaagcacaaacacgctaagtgccggccgtccgagcgcacgcagcagcaaggctgcaacgttggccagcctggcagacacgccagccatgaagcgggtcaactttcagttgccggcggaggatcacaccaagctgaagatgtacgcggtacgccaaggcaagaccattaccgagctgctatctgaatacatcgcgcagctaccagagtaaatgagcaaatgaataaatgagtagatgaattttagcggctaaaggaggcggcatggaaaatcaagaacaaccaggcaccgacgccgtggaatgccccatgtgtggaggaacgggcggttggccaggcgtaagcggctgggttgtctgccggccctgcaatggcactggaacccccaagcccgaggaatcggcgtgagcggtcgcaaaccatccggcccggtacaaatcggcgcggcgctgggtgatgacctggtggagaagttgaaggccgcgcaggccgcccagcggcaacgcatcgaggcagaagcacgccccggtgaatcgtggcaagcggccgctgatcgaatccgcaaagaatcccggcaaccgccggcagccggtgcgccgtcgattaggaagccgcccaagggcgacgagcaaccagattttttcgttccgatgctctatgacgtgggcacccgcgatagtcgcagcatcatggacgtggccgttttccgtctgtcgaagcgtgaccgacgagctggcgaggtgatccgctacgagcttccagacgggcacgtagaggtttccgcagggccggccggcatggccagtgtgtgggattacgacctggtactgatggcggtttcccatctaaccgaatccatgaaccgataccgggaagggaagggagacaagcccggccgcgtgttccgtccacacgttgcggacgtactcaagttctgccggcgagccgatggcggaaagcagaaagacgacctggtagaaacctgcattcggttaaacaccacgcacgttgccatgcagcgtacgaagaaggccaagaacggccgcctggtgacggtatccgagggtgaagccttgattagccgctacaagatcgtaaagagcgaaaccgggcggccggagtacatcgagatcgagctagctgattggatgtaccgcgagatcacagaaggcaagaacccggacgtgctgacggttcaccccgattactttttgatcgatcccggcatcggccgttttctctaccgcctggcacgccgcgccgcaggcaaggcagaagccagatggttgttcaagacgatctacgaacgcagtggcagcgccggagagttcaagaagttctgtttcaccgtgcgcaagctgatcgggtcaaatgacctgccggagtacgatttgaaggaggaggcggggcaggctggcccgatcctagtcatgcgctaccgcaacctgatcgagggcgaagcatccgccggttcctaatgtacggagcagatgctagggcaaattgccctagcaggggaaaaaggtcgaaaacactctttcctgtggatagcacgtacattgggaacccaaagccgtacattgggaaccggaacccgtacattgggaacccaaagccgtacattgggaaccggtcacacatgtaagtgactgatataaaagagaaaaaaggcgatttttccgcctaaaactctttaaaacttattaaaactcttaaaacccgcctggcctgtgcataactgtctggccagcgcacagccgaagagctgcaaaaagcgcctacccttcggtcgctgcgctccctacgccccgccgcttcgcgtcggcctatcgcggccgctggccgctcaaaaatggctggcctacggccaggcaatctaccagggcgcggacaagccgcgccgtcgccactcgaccgccggcgcccacatcaaggcaccctgcctcgcgcgtttcggtgatgacggtgaaaacctctgacacatgcagctcccggagacggtcacagcttgtctgtaagcggatgccgggagcagacaagcccgtcagggcgcgtcagcgggtgttggcgggtgtcggggcgcagccatgacccagtcacgtagcgatagcggagtgtatactggcttaactatgcggcatcagagcagattgtactgagagtgcaccatatgcggtgtgaaataccgcacagatgcgtaaggagaaaataccgcatcaggcgctcttccgcttcctcgctcactgactcgctgcgctcggtcgttcggctgcggcgagcggtatcagctcactcaaaggcggtaatacggttatccacagaatcaggggataacgcaggaaagaacatgtgagcaaaaggccagcaaaaggccaggaaccgtaaaaaggccgcgttgctggcgtttttccataggctccgcccccctgacgagcatcacaaaaatcgacgctcaagtcagaggtggcgaaacccgacaggactataaagataccaggcgtttccccctggaagctccctcgtgcgctctcctgttccgaccctgccgcttaccggatacctgtccgcctttctcccttcgggaagcgtggcgctttctcatagctcacgctgtaggtatctcagttcggtgtaggtcgttcgctccaagctgggctgtgtgcacgaaccccccgttcagcccgaccgctgcgccttatccggtaactatcgtcttgagtccaacccggtaagacacgacttatcgccactggcagcagccactggtaacaggattagcagagcgaggtatgtaggcggtgctacagagttcttgaagtggtggcctaactacggctacactagaaggacagtatttggtatctgcgctctgctgaagccagttaccttcggaaaaagagttggtagctcttgatccggcaaacaaaccaccgctggtagcggtggtttttttgtttgcaagcagcagattacgcgcagaaaaaaaggatctcaagaagatcctttgatcttttctacggggtctgacgctcagtggaacgaaaactcacgttaagggattttggtcatgGtTCTGAGATTATCAAAAAGGATCTTCACCTAGATCCTTTTAAATTAAAAATGAAGTTTTAAATCAATCTAAAGTATATATGTGTAACATTGGTCTAGTGATTAGAAAAACTCATCGAGCATCAAATGAAACTGCAATTTATTCATATCAGGATTATCAATACCATATTTTTGAAAAAGCCGTTTCTGTAATGAAGGAGAAAACTCACCGAGGCAGTTCCATAGGATCGCAAGATCCTGGTATCGGTCTGCGATTCCGACTCGTCCAACATCAATACAACCTATTAATTTCCCCTCGTCAAAAATAAGGTTATCAAGTGAGAAATCACCATGAGTGACGACTGAATCCGGTGAGAATGGCAAAAGTTTATGCATTTCTTTCCAGACTTGTTCAACAGGCCAGCCATTACGCTCGTCATCAAAATCACTCGCATCAACCAAACCGTTATTCATTCGTGATTGCGCCTGAGCGAGACGAAATACGCGGTCGCTGTTAAAAGGACAATTACAAACAGGAATCGAATGCAACCGGCGCAGGAACACTGCTAGCGCATCAACAATATTTTCACCGCTATCCGGATATTCTTCTAATACCTGGAATGCTGTTTTTCCGGGGATCGCAGTGGTGAGTAACCATGCATCATCAGGAGTACGGATAAAATGCTTGATGGTCGGAAGAGGCATAAATTCCGTCAGCCAGTTTAGTCTGACCATCTCATCTGTAACATCATTGGCAACGCTACCTTTGCCATGTTTCAGAAACAACTCTGGCGCATCGGGCTTCCCATACAAGCGATAGATTGTCGCACCTGATTGCCCGACATTATCGCGAGCCCATTTATACCCATATAAATCAGCATCCATGTTGGAATTTAATCGCGGCCTGCTGCAAGACGTTTCCCGTTGAATATGGCTCATAACACCCCTTGTATTACTGTTTATGTAAGCAGACAGTTTTATTGTTCATGATGATATATTTTTATCTTGTGCAATGTAACATCAGAGATTTTGAGACACAACGTGGCTTTCGGAAcatgatatatctcccaatttgtgtagggcttattatgcacgcttaaaaataataaaagcagacttgacctgatagtttggctgtgagcaattatgtgcttagtgcatctaacgcttgagttaagccgcgccgcgaagcggcgtcggcttgaacgaatttctagctagacattatttgccgactaccttggtgatctcgcctttcacgtagtggacaaattcttccaactgatctgcgcgcgaggccaagcgatcttcttcttgtccaagataagcctgtctagcttcaagtatgacgggctgatactgggccggcaggcgctccattgcccagtcggcagcgacatccttcggcgcgattttgccggttactgcgctgtaccaaatgcgggacaacgtaagcactacatttcgctcatcgccagcccagtcgggcggcgagttccatagcgttaaggtttcatttagcgcctcaaatagatcctgttcaggaaccggatcaaagagttcctccgccgctggacctaccaaggcaacgctatgttctcttgcttttgtcagcaagatagccagatcaatgtcgatcgtggctggctcgaagatacctgcaagaatgtcattgcgctgccattctccaaattgcagttcgcgcttagctggataacgccacggaatgatgtcgtcgtgcacaacaatggtgacttctacagcgcggagaatctcgctctctccaggggaagccgaagtttccaaaaggtcgttgatcaaagctcgccgcgttgtttcatcaagccttacggtcaccgtaaccagcaaatcaatatcactgtgtggcttcaggccgccatccactgcggagccgtacaaatgtacggccagcaacgtcggttcgagatggcgctcgatgacgccaactacctctgatagttgagtcgatacttcggcgatcaccgcttcccccatgatgtttaactttgttttagggcgactgccctgctgcgtaacatcgttgctgctccataacatcaaacatcgacccacggcgtaacgcgcttgctgcttggatgcccgaggcatagactgtaccccaaaaaaacagtcataacaagccatgaaaaccgccactgcgccgttaccaccgctgcgttcggtcaaggttctggaccagttgcgtgagcgcatacgctacttgcattacagcttacgaaccgaacaggcttatgtccactgggttcgtgcccgaattgatcacaggcagcaacgctctgtcatcgttacaatcaacatgctaccctccgcgagatcatccgtgtttcaaacccggcagcttagttgccgttcttccgaatagcatcggtaacatgagcaaagtctgccgccttacaacggctctcccgctgacgccgtcccggactgatgggctgcctgtatcgagtggtgattttgtgccgagctgccggtcggggagctgttggctggctggtggcaggatatattgtggtgtaaacaaattgacgcttagacaacttaataacacattgcggacgtttttaatgtactgaattaacgccgaattgaattatcagcttgcatgccggtcgatctagtaacatagatgacaccgcgcgcgataatttatcctagtttgcgcgctatattttgttttctatcgcgtattaaatgtataattgcgggactctaatcataaaaacccatctcataaataacgtcatgcattacatgttaattattacatgcttaacgtaattcaacagaaattatatgataatcatcgcaagaccggcaacaggattcaatCTTAAGAAACTTTATTGCCAAATGTTGAACGATGGATCAAATCTCGGTGACTGGCAGGACAGGACGTGGCGGCACCGGCAGGCTGAAGTCCAGCTGCCAGAAACCCACGTCATGCCAGTTCCCGTGCTTGAAGCCGGCCGCCCGCAGCATGCCGCGGGGGGCATATCCGAGCGCCTCGTGCATGCGCACGCTCGGGTCGTTGGGCAGCCCGATGACAGCGACCACGCTCTTGAAGCCCTGTGCCTCCAGGGACTTCAGCAGGTGGGTGTAGAGCGTGGAGCCCAGTCCCGTCCGCTGGTGGCGGGGGGAGACGTACACGGTCGACTCGGCCGTCCAGTCGTAGGCGTTGCGTGCCTTCCAGGGGCCGGCGTAGGCGATGCCGGCGACCTCGCCGTCCACCTCGGCGACGAGCCAGGGATAGCGCTCCCGCAGACGGACGAGGTCGTCCGTCCACTCCTGCGGTTCCTGCGGCTCGGTACGGAAGTTGACCGTGCTTGTCTCGATGTAGTGGTTGACGATGGTGCAGACCGCCGGCATGTCCGCCTCGGTGGCACGGCGGATGTCGGCCGGGCGTCGTTCTGGGCTCATGGTTACTTCCTAATCTATGGTTCCGCGATAATTGTAAATGTAATTGTAATGTTGTTTGTTGTTTGTTGTTGTTGGTAATTGTTGTAAAAATGCTTATACTCCAGCGTGTCCTCTCCAAATGAAATGAACTTCCTTATATAGAGGAAGGGTCTTGCGAAGGATAGTGGGATTGTGCGTCATCCCTTACGTCAGTGGAGATGTCACATCAATCCACTTGCTTTGAAGACGTGGTTGGAACGTCTTCTTTTTCCACGATGCTCCTCGTGGGTGGGGGTCCATCTTTGGGACCACTGTCGGCAGAGAGATCCTGCGAAGGATAGTGGGATTGTGCGTCATCCCTTACGTCAGTGGAGATGTCACATCAATCCACTTGCTTTGAAGACGTGGTTGGAACGTCTTCTTTTTCCACGATGCTCCTCGTGGGTGGGGGTCCATCTTTGGGACCACTGTCGGCAGAGAGATCCTGCGAAGGATAGTGGGATTGTGCGTCATCCCTTACGTCAGTGGAGATGTCACATCAATCCACTTGCTTTGAAGACGTGGTTGGAACGTCTTCTTTTTCCACGATGCTCCTCGTGGGTGGGGGTCCATCTTTGGGACCACTGTCGGCAGAGAGATCCTGCGAAGATAGTGGGATTGTGCGTCATCCCTTACGTCAGTGGAGATGTCACATCAATCCACTTGCTTTGAAGACGTGGTTGGAACGTCTTCTTTTTCCACGATGCTCCTCGTGGGTGGGGGTCCATCTTTGGGACCACTGTCGGCA

1. Map of the pDW3876 construct (dual *Bsa*I cut sites for insertion of gRNA are highlighted in blue)
